# Metabolism of energy substrates of *in vitro* and *in vivo* derived embryos from ewes synchronized and super ovulated with norgestomet and porcine follicle stimulating hormone

**DOI:** 10.1186/2049-1891-3-37

**Published:** 2012-11-17

**Authors:** Ahmad Azizi-Moghadam

**Affiliations:** 1 Department of Clinical Sciences, College of Veterinary Medicine, University of Zabol, Zabol, 98613635856, Iran

**Keywords:** Crestar, Glucose, Lactate, Metabolism, Norgestomet, Pyruvate

## Abstract

The synchronization and ovulatory responses of Sangsari cross bred ewes and metabolism of energy substrates in 8-cell stage embryos to hatched blastocysts stage produced *in vitro* or *in vivo* were investigated. Ewes were assigned randomly to receive 37.5 IU of porcine follicle stimulating hormone (FSH-P) daily for the 3 days preceding implant removal (Day 0). Synchronization of estrus was carried out using a 1.5 mg norgestomet (Crestar) ear implant for 12 days. Ewes in estrus were mated two to three times with rams of proven fertility. At the time of first mating each ewe was administered 1000 IU of human chorionic gonadotrophin (hCG) to induce ovulation. Surgical embryo recovery was performed on Days 4 and 6 after onset of estrus (Day 0) and recovered embryos were subjected to comparative metabolism studies with *in vitro* derived embryos at the same stage of development. The number of corpora lutea (CL), unovulated follicles and overall ovarian activity were recorded for each ewe during the breeding and non-breeding seasons. While the pattern of oxidation was similar among *in vitro* and *in vivo* derived embryos, a low pyruvate to lactate ratio was the preferred substrate of embryos derived *in vitro*. A high level of production of CO2 and lactate resulted from a stress response to the suboptimal culture environment. The first marked increase in the metabolism of glucose by ovine embryos was detected in compact morula stage, but there was no significant increase in the oxidation of glucose after the morula stage. Two different concentrations of glucose were compared, but this did not affect metabolism. However, the rate of incorporation and metabolism of glucose tended to be higher at the 0.56 mmol/L glucose dosage.

## Introduction

Synchronization of estrus followed by induction of ovulation are basic techniques in all embryo transfer programs. Superovulation ensures recovery of multiple embryos during one estrous cycle, thus allowing us to maximally exploit the female germ pool. Reports of synchronization of estrus and superovulation of ewes have been previously discussed [1,2]. The production of CO2 from glucose by sheep embryos has been determined [3] and results have indicated that little glucose is utilized by the embryos of sheep and that blastocysts derived from oocytes, matured and fertilized *in vitro*, oxidized glucose at a lower rate than those developed *in vivo*. In addition, pyruvate (0.33 mmol/L) and lactate (3.3 mmol/L) together were beneficial to early embryonic development in sheep [3]. Rieger [4] inferred that the pre-implantation bovine embryo undergoes an intensified period of cellular synthetic processes which require appropriate substrates for production of energy and reducing equivalents in the form of nicotinamide-adenine dinucleotide phosphate. The objective of this experiment was to evaluate the effectiveness of Crestar for synchronization of estrus and porcine Follicle Stimulating Hormone (FSH-P) in inducing superovulation of ewes and to compare metabolism of *in vitro* and *in vivo* derived embryos from the 8-cell to hatched blastocyst stages Enhancing the understanding of metabolic activity of pre implantation embryos with respect to improving the yield and quality of embryos was also an objective of this study.

## Materials and methods

### Animals

A total of 23 ewes (Sangsari cross bred) between 2 and 5 years of age that were subjected to standard feeding and management protocols were used. Ewes were isolated from rams except at the time of mating. After deworming, ewes were tagged for identification and assigned randomly to either be bred during the normal breeding season (October through December; n = 12 ewes) or out of season (June through August, n = 11 ewes). All animal experiments were approved by the Zabol University and Semnan Province Agricultural Jihad sheep breeding farm Animal Ethics Committee in accordance with the care and use of animals for scientific purposes follow internationally recognized guidelines, approved by the Iranian higher education and research ministry.

### Estrus synchronization and superovulation protocols

Synchronization of estrus was achieved by insertion of a 1.5 mg norgestomet ear implant (17- α-aceto-11β methyl_−_ 19- nor_−_ preg-4-en-3,20 dione) (Crestar™ Intervet International, Boxmeer, The Netherlands) that remained in place for 12 days. Prior to removal of the progestagen ear implant, each ewe was treated twice daily at 12 h intervals with FSH-P (Super- Ov-; Ausa International, USA) with 16 IU, 12 IU and 9.5 IU on Day -2, Day -1 and Day 0. Day 0 was the day the Crestar implant was removed. Beginning at 8 h after implant removal, ewes were observed for estrus three times each day, both visually and in the presence of teaser rams. When detected in estrus, ewes were mated two to three times with fertile rams. At the time of the first mating, each ewe was administered 1000 IU hCG (Chorulon; Intervet International; to induce ovulation.

### Flushing embryos from oviduct and uterus

**Figure 1:**
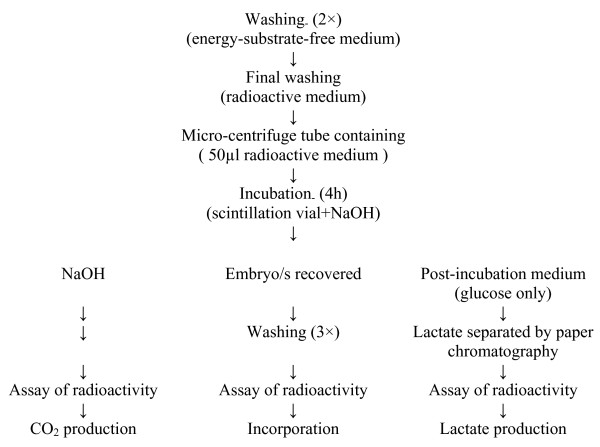
A schematic representation of the procedure followed for estimating metabolic activity of embryos and blastocysts.

All ewes in the breeding and nonbreeding season were subjected to surgery to allow the oviducts and uterine horns to be flushed on Day 4 (n = 6 for breeding season and n = 5 for non-breeding season) or Day 6 (n = 6 for breeding season and n = 6 for non-breeding season) using Dulbecco’s Phosphate Buffer Saline (ZT156; IMV, France) as the flushing medium. On Day 4 each oviduct and uterine horn was flushed, whereas, only the uterine horns were flushed on Day 6 post mating. The ovarian response of each ewe was recorded with respect to the number of corpora lutea, unovulated follicles and overall ovarian activity as described previously [5] ( Figure 1).

### Embryo metabolism

Catabolic metabolism was evaluated by measuring the production of CO2 from three substrates (lactate, pyruvate and glucose) and lactate production from glucose was also determined. Anabolic metabolism was estimated by determining the amount of substrate carbon incorporated by the embryos. A specially washed and heat sterilized 20 mL scintillation vial served as the incubation apparatus. Embryos were washed through two changes (2 mL) of energy-substrate-free PBS and placed in a 50 μL drop of radioactive medium for a final wash before being transferred into a sterile, 600 μL micro centrifuge tube (Eppendorf Netheler Hinz; Gmbh, Hamburg, Germany) containing 50 μL radioactive incubation medium. This tube was placed in the scintillation vial with the lid of the tube acting as a support. Simultaneously, 0.5 mL of 2.5 mol/L sodium hydroxide was added to the bottom of the vial, which was then closed and incubated at 39°C for 4 h. At the end of the incubation period, the tube with embryos was taken out and the radioactivity trapped as CO2 was determined after the addition of scintillation cocktail. In all experiments, at least two sham incubations were run concurrently to correct for background counts. The metabolic turnover was estimated from the disintegrations per minute detected in the sample and the specific activity of the parent substrate, and converted to picomoles/embryo/h on the basis that each glucose molecule gives rise to 6 molecules of CO2 [6]. Oocytes surrounded by about three layers of compact cumulus oophorous cells were obtained by puncturing ovarian follicles of more than 10 mm diameter and placed in culture medium (DPBS), maturation medium (TCM-199 Gibco Lab, New York;- Earls salt, Centron Research Lab, India), and then the culture medium was modified by the addition of sodium pyruvate 22 ug/mL, sodium bicarbonate 2.2 mg/mL and streptomycin sulphate 0.5 mg/mL to prevent fungus formation in the medium. Then BMOC-2 (pH 7.4) and medium were added for sperm capacitation and fertilization_−_ (Freshly prepared modified Bracketts defined medium without glucose [7]-, along with addition of proper additives (10% -v/v) heat-inactivated fetal calf serum (FCS; Bio-Lab, Tehran,), ovine LH10 mg/mL(NIADDK-NIH-26, AFP5551B; Bethesda, MD), FSH1 mg/mL (NIADDK-NIH-20, AFP7028D; Bethesda, MD) were used for oocytes IVF and IVM.

### Comparative metabolic studies of embryos

The collected oocytes first were fertilized and developed up to the 8-cell stage *in vitro*, then subjected to comparative metabolism studies with in vivo derived embryos at the same stage of development for assessment of energy differences and requirement for further development up to hatched blastocyst stage. The *in vitro* development of *in vivo* and *in vitro* derived embryos was assessed at 4 h intervals. The metabolism of 8- to 16- cell stage embryos up to the hatched blastocyst stage was investigated using either glucose, pyruvate or lactate as the sole energy substrate. Surgically recovered embryos at the 8-cell to morula and blastocyst stages and derived *in vivo* were subjected to comparative metabolism of energy substrate with embryos at the same stage derived *in vitro*.

### Classification of recovered embryos

Recovered embryos were classified according to International Embryo Transfer Society (IETS) guidelines [8], and washed individually four times in fresh flushing medium. Excellent and good quality embryos were used in metabolic studies that were carried out in four parts as follows:

1) Eight-cell embryos were used to assess metabolism before the so- called block to development stages prior to activation of the embryonic genome [9].

2) Morula stage embryos obtained on Day 6 of culture were used to elucidate changes in metabolism after the block to development and before compaction of blastomeres and the period of rapid growth to the blastocyst stage.

3) Blastocysts obtained on Days 7 to 9 post-mating were classified as early- and mid-stage blastocysts and expanded blastocysts before being used for metabolic studies. The overall diameter of blastocysts, including the zona pellucida, was measured with an ocular micrometer at two axes perpendicular to each other and expressed as the mean of the two measurements. These measurements were made before metabolic studies were performed to find relationships between size and metabolic activity. The *in vivo* derived early-stage blastocysts were cultured *in vitro* for 24 or 48 h to obtain mid-stage and expanded blastocysts, respectively.

4) Hatching or hatched blastocysts were obtained on Days 8 to 10 of culture and subjected to metabolic studies to assess changes during the process of hatching and post-hatching. *In vivo* derived early blastocysts were grown *in vitro* for 24 h to obtain hatched blastocysts.

### Statistical analyses

The data obtained from all experiments were subjected to appropriate statistical analyses as described previously [10]. In the text, the data are presented as means ± se. Differences in the frequencies of maturation, fertilization and cleavage and the proportion of the zygotes reaching the morula and blastocyst stages were analyzed by the Chi-square test. Similarly, the data obtained for CO2 production by the embryos before and after cryopreservation were subjected to the Students t-test. Comparison of multiple means was performed by the protected least significant difference test. In all statistical analyses when the probability of an effect being due to chance was less than (P < 0.05), the effect was considered to be statistically significant.

## Results and discussion

Irrespective of season of the year, the majority of ewes exhibited estrus 24 h following implant removal and in the non-breeding season only 4 ewes exhibited estrus at 48 h after implant removal (Table 1). The mean interval to estrus and the duration of estrus were similar to those reported by Morton and Maxwell [2]. A super-ovulatory response was obtained during both the breeding season and non-breeding season (Table 2 and Table 3), but the response was slightly lower in the nonbreeding season which is similar to results reported previously [2]. This previous report [2] indicated that ovulation rates were significantly higher with the decreasing dose schedule of FSH during the breeding season. The present study also used Super-Ov with the decreasing dose rate as reported previously [11]. The comparative data during the nonbreeding season indicated a somewhat lower number of ovulations for the individual ovaries and as a total ovulatory response for both ovaries when compared to results from ewes in the breeding season (Table 2 and Table 3). The size of unovulated follicles during breeding season was very large, so that they resembled cystic follicles (diameter > 15 mm).

**Table 1:** Occurrence of estrus in ewes bred following super ovulation

**S. No**	**Season**	**No. of Donors**	**Hours to estrus following implant withdrawal**					**Interval to estrus(h)**	**Duration of estrus (h)**
			**12**	**24**	**36**	**48**	**60**		
1	Non-breeding	11	0	7	0	4	0	32.00 ± 8.00	18.55 ± 1.27
2	Breeding	12	0	12	0	0	0	24.00 ± 0.00	21.00 ± 2.05

**Table 2:** Ovarian response of ewes following superovulation during the breeding season

**Number of animals**			**Corpora lutea**			**Unovulated follicles**			**Overall ovarian activity**
FSH-P Treated	Right Ovary	Left Ovary	Total	Right Ovary	Left Ovary	Total	Right Ovary	Left Ovary	Total
12	18	40	58	12	2	14	30	42	72
Mean ± se	1.50 ± 0.34	3.33 ± 0.42	4.83 ± 0.60	1.00 ± .26	0.17 ± .17	1.17 ± 0.31	2.50 ± .43	3.50 ± .34	6.00 ± 0.45

Mean ± se total ovulation response, mean ± se total unovulated follicles the mean ± se total overall ovarian activity in the breeding season.

**Table 3:** Ovarian response of ewes following superovulation in the non-breeding season

**Number of animals**			**Corpora lutea**			**Unovulated follicles**			**Overall ovarian activity**
FSH-P Treated	Right Ovary	Left Ovary	Total	Right Ovary	Left Ovary	Total	Right Ovary	Left Ovary	Total
11	16	28	44	9	4	13	25	32	57
Mean ± se	1.45 ± 0.21	2.55 ± 0.34	4.00 ± 0.47	0.82 ± .33	0.36 ± .15	1.18 ± 0.44	2.27 ± .38	2.91 ± .37	5.18 ± 0.64

Mean ± se total ovulation response, mean ± se total unovulated follicles and mean ± se total overall ovarian activity in the non-breeding season.

It has been reported that ear implants containing 3 mg progesterone offer a reliable means for synchronization of estrus in ewes [12], whereas the use of only 1.5 mg norgestomet in combination with FSH-E and GnRH at first mating for both synchronization of estrus and induction of ovulation induced a greater superovulatory response [5]. As summarized in (Table 2), breed (crossbred), hormone used for inducing superovulation(FSH-E), and differences in effects induced by GnRH affect the ovulatory response. In contrast, there has been an attempt to minimized variation in time of ovulation using either GnRH or hCG [13]. The most important finding from this study is related to the uniformity in the percentage of ovulating follicles when ewes were given 1000 IU hCG at the time of first mating. However, there is the unanswered question of whether a similar rate of ovulation would have been obtain if hCG was not administered, but results of the present study support previous findings [13] although further research is required. During the breeding season, 58 ovulations occurred. When the oviducts and uterine horns were flushed on Day 4 post-estrus, 18 embryos were recovered, of which 16 were morphologically normal and two were abnormal and discarded. On Day 4 post-estrus only 30 ovulations occurred. When uterine flushing was carried out on Day 6 post-estrus, 17 embryos were recovered and all were morphologically normal. On Day 6 post-estrus only 28 ovulations occurred. During the non-breeding season, a total of 44 ovulations occurred. When the oviducts and uterine horns were flushed on Day 4 post-estrus only 9 embryos at the 4-cell stage were recovered. On Day 4 post-estrus only 31 ovulations occurred. But, when only uterine flushing carried out on Day 6 post-estrus, three embryos were recovered out of 13 ovulations, and all embryos were morphologically normal. These studies were conducted to observed differences between breeding and nonbreeding seasons. The retarded or degenerated embryos could have resulted from either late ovulations or an arrest in their developmental process. However, if they were morphologically normal, such retarded embryos may have the potential to develop further if provided a suitable uterine environment [14]. These results are similar to those reported earlier suggesting that high estrogen output from large unovulated follicles speeds up the rate of transport, culminating in low rates of embryo recovery [15,16]. On Day 6 one expects to find early, late and expanded blastocysts, and this was the situation here. It has been suggested that somatic cells facilitate growth of embryos by reducing O2 tension and/or increasing concentrations of CO2 in the vicinity of embryos [17]. Based on the reports from research with several other species including rat, rabbit and monkey [18], the intra-uterine O2 concentration appears to be in the range of 1.5 to 7%.

### Flushing of uterine horns and oviducts

Flushing the uterine horns and oviducts on Day 4 post mating during the breeding and nonbreeding seasons yielded embryos with developmental stages varying between 8-cells to morula, with, two embryos classified as degenerated, but the remaining embryos had normal morphology and were considered as transferable to a recipient ewe. Flushing only uterine horns on Day 6 during the breeding and non-breeding seasons yielded embryos varying from the 16-cell to hatched blastocyst stages and all had normal morphology and were considered as transferable to a recipient ewe.

### Analysis of embryo quality

An overall analysis of embryos quality, irrespective of season, revealed that morulae and blastocysts were recovered only from ewes flushed on Day 6 post-estrus as expected. The embryos were classified according to International Embryo Transfer Society (IETS) guidelines [8], washed individually four times in fresh flushing medium and then placed in holding medium (Dulbecco’s phosphate buffered saline containing 0.4% w/v bovine serum albumin). Only excellent and good embryos were selected for metabolism studies.

### *In vitro* metabolism of energy substrates

The *in vitro* metabolism of energy substrates by 8-cell stage embryos derived *in vivo* was compared to that for the same stage embryos derived *in vitro*. The pattern of oxidation was similar between the two types of embryos, but oxidation and CO2 production and lactate production was greater for *in vitro* as compared to *in vivo* derived 8-cell stage embryos (c versus d = (p < 0.05). In general the two concentrations of glucose did not differ in their metabolic fate, although the rate of glucose incorporation tended to be higher in the presence of 0.56 mmol/L glucose. However, production of lactate and uptake of glucose carbon were higher in the presence of 0.56 mmol/L glucose. No lactate production was detected apart from the higher oxidation of lactate by the *in vivo* derived embryos of both stages (Table 4). The *in vivo* collected morulae and early blastocysts produced lower rates of CO2 (p < 0.05) than their counterparts derived *in vitro*. Similarly, the mid-stage blastocysts cultured for 24 h produced more CO2 as compared to values for freshly collected and cultured mid-stage blastocysts. In general, lactate production by the freshly obtained embryos was about one half that for *in vitro* derived embryos. However, long pre-exposure to culture conditions narrowed the differences between the two sources at the hatched blastocyst stage (72 h of IVC) and were not significant. Incorporation of glucose carbon was independent of the source of embryos, but there was a tendency for a lower uptake by IVM/IVF embryos from mid stage to hatched blastocyst stages (Table 5). The concentration of glucose (0.28 mmol/L) used in the bulk of the present experiments to study embryo metabolism is close to that observed in bovine oviductal fluid [18]. Similarly, the concentration of lactate (2–5 mmol/L) seems to be in the physiological range based on reported values for sheep and rabbit oviductal fluid [15].

**Table 4:** **Metabolism of energy substrate by 8- cell embryos that were derived *in vitro *or *in vivo***

**Energy substrate**	***In Vitro***	***In Vivo***
**(a)Productionof CO2(p moles)**		
Pyruvate	2.52 ± 0.26	2.20 ± 0.46
Lactate	2.30 ± 0.49^c^	0.50 ± 0.8^d^
Glucose		
0.28 mmol/L	0.29 ± 0.10	0.19 ± 0.05
0.56 mmol/L	0.36 ± 0.80	NP
**(b)Incorporation(pg atoms)**		
Pyruvate	1.27 ± 0.04	1.30 ± 0.93
Lactate	1.53 ± 0.38	0.76 ± 0.13
Glucose		
0.28 mmol/L	3.00 ± 0.70^a^	4.50 ± 0.24^b^
0.56 mmol/L	4.72 ± 0.59^b^	NP

Values are means ± SEM (per embryo per hour) for five replicates;

Means with different superscripts (a,b,c,d) in the each row are different significantly(p < 0.05):

NP: not performed.

**Table 5:** **Comparative metabolism of glucose by embryos produced *in vitro *(IVM/IVF), obtained by flushing the uterus and used immediately (fresh) or obtained from the uterus and cultured *in vitro *for 1–3 days (fresh + IVC)**

**Biochemical parameter/source of embryo**	**Morula**	**Stages early blastocyst**	**Development Mid blastocyst**	**Blastocyst**	**Hatched blastocyst**
			**(a)Catabolic**		
			**Utilization (Ι)**		
			**CO**_**2**_		
			**(p moles/h)**		
IVM/	7.00 ± 1.50^a^	8.21 ± 1.90^a^	8.00 ± 0.12	9.80 ± 1.50	10.65 ± 1.40
Fresh	3.00 ± 0.55^b^	4.15 ± 0.21^b^	7.20 ± 0.50^a^	NP	NP
Fresh+IVC	NP	NP	10.13 ± 0.38^b^	9.24 ± 0.32	10.35 ± 0.75
			**(ΙΙ)Lactate**		
			**Production**		
			**(p moles/h)**		
IVM/IVF	24.80 ± 5.57^a^	26.00 ± 6.30^a^	43.94 ± 7.60^b^	69.14 ± 4.50^a^	66.14 ± 8.63
Fresh	10.82 ± 1.40^b^	10.02 ± 1.43^b^	20.60 ± 5.97^a^	NP	NP
Fresh+IVC	NP	NP	38.54 ± 1.55^b^	39.52 ± 8.65^b^	54.13 ± 20.94
			**(b)Incorporation**		
			**(p mole/h)**		
IVM/IVF	17.70 ± 4.13	18.00 ± 5.07	14.92 ± 2.04^a^	21.71 ± 4.52	21.88 ± 2.01
Fresh	13.66 ± 1.76	15.50 ± 0.73	17.00 ± 0.58^a^	NP	NP
Fresh+IVC	NP	NP	43.43 ± 10.67^b^	27.96 ± 3.70	30.21 ± 9.96

Values are means ± SEM (per embryo per h); NP: not performed a,b differ significantly within biochemical parameter in each row or column of a stage of development (p < 0.05).

The concentration of pyruvate was the same as used previously for mouse embryos [19]. The results of the initial experiments for determining the rate of metabolism of glucose up to late preimplantation stages is similar to that reported for sheep embryos [20]. The first marked increase in the metabolism of glucose by bovine embryos occurs between the 8 to 16-cell stage and oxidation of glucose does not differ between 8- and 12-cell stage embryos [4]. A higher rate of total glucose metabolism was detected for compacted morulae, but there was no significant increase in the oxidation of glucose after the morula stage, although the number of cells almost doubled between the morula and early blastocyst stages [21]. The formation of tight junctions at the morula stage may require more energy and other substances derived from glucose. In general, the first significant increase in the metabolism of glucose occurred after activation of the embryonic genome and the second rise is associated with the formation of the blastocoelic activity, probably to meet energy demands of the ion pump involved in the fluid accumulation [22]. The expanding blastocysts obtained after 24 h culture of fresh early-stage blastocysts recovered from the uterus had higher rates of O2 oxidation as reported previously [22]. The most surprising feature of glucose metabolism by *in vitro* and *in vivo* derived embryos was the lack of detectable lactate production until the 12- cell stage. When lactate was detected its production increased continuously through the blastocyst stage. *In vivo* derived embryos produced one-half the quantity of lactate as *in vitro* derived embryos. Lactate production by embryos recovered by flushing the oviduct and uterus was also about one-half that for *in vitro* derived embryos. However, this difference decreased as time in culture increased.

## Conclusion

Season and time of onset of estrus did not affect the ultimate number of transferable embryos in ewes. However, results of this study indicate: that ewes exhibit a lower ovulatory response compared with results reported for crossbred ewes in; a previous study [23]. In the presence of exogenous hCG, endogenous LH was apparently sufficient to cause ovulation but it was not determined if similar rates of ovulation would have been obtained if hCG was not administered. Metabolism studies of radiolabeled pyruvate and lactate indicated that pyruvate was the preferred substrate up to the 12 cell-stage and its oxidation increased at during compaction and blastulation of morulae. Therefore, results of the present study suggest that a low pyruvate to lactate ratio should be beneficial during culture of ovine embryos from the 12-cell stage to the hatched blastocyst stage in chemically defined media. One of the explanations for high lactate production could be a stress response to a suboptimal culture environment. The culture environment that provided optimum development was due to variability in the pH and oxygen tension, which in this experiment could not be maintained constantly throughout the experiment.

## Competing interests

Dr Ahmad Azizimoghadam declare that they have no competing interests.

## Author contribution

Dr AA carried out the molecular genetic studies, participated in the sequence alignment and drafted the manuscript. The author read and approved the final manuscript.
